# The effects of a cluster-randomized control trial manipulating exercise goal content and planning on physical activity among low-active adolescents

**DOI:** 10.3389/fpsyg.2022.950107

**Published:** 2022-09-14

**Authors:** Damien Tessier, Virginie Nicaise, Philippe Sarrazin

**Affiliations:** ^1^Univ. Grenoble Alpes, SENS, Grenoble, France; ^2^Laboratory of Vulnerabilities and Innovation in Sport, University of Claude Bernard Lyon 1, Univ Lyon, Villeurbanne, France

**Keywords:** framing message, self-regulation, physical activity, self-determination theory, theory of planned behavior

## Abstract

The purpose of the present two studies was to investigate whether in framing messages that target salient beliefs of youth, the type of goal framed matter to promote physical activity (PA) participation among low-active adolescents (i.e., participating in less than 1 h/day of moderate-to-vigorous PA). More specifically, the main trial (study 2) compared the effect of intrinsic and extrinsic-goal framing messages alongside planning (IMC + P and EMC + P) to a control condition (CC) on low-active adolescents’ physical activity (PA), intention, attitude, and exercise goals, and examined the potential meditational effect of these variables between condition and PA. Low-active students (*n* = 193; *M* age = 16.89) from fifteen classes were assigned to one of these three conditions. PA was assessed using an accelerometer, and the socio-cognitive mediators were measured at baseline (i.e., 2 weeks before the intervention) and post-test, and the intention was measured again at follow-up (i.e., 2 weeks after the intervention). Results showed that compared to adolescents in the CC group, those in the experimental conditions did not do more moderate PA, but carried out more light PA, and yielded an increase in attitude and intention. Mediational analysis revealed no significant effect of the potential mediators.

## Introduction

Despite the fact that participation in regular physical activity (PA) has numerous health benefits for young people [e.g., lower incidence of a broad variety of chronic diseases, and generating positive mental health outcomes; for more details see Hallal et al. (2006), Biddle and Asare (2011)], many children and adolescents are physically inactive and become even less active as they become older. Self-reported PA collected across 105 countries reveals that 80% of youths aged 13–15 years old do not reach the recommended public health guidelines of 60 min PA per day (World Health Organization, 2009, 2010, 2011); for a review, see Hallal et al. (2012), Inchley et al. (2016). This rate is even lower when PA is measured with objective measures (i.e., accelerometers). For example, a study of 10 to 12-year-old European children reported that only 4.6% of girls and 16.8% of boys achieved these recommendations (Verloigne et al., 2012). These percentages are alarming and imply an urgent need to develop effective theory-based PA promotion programs for low-active adolescents (Gourlan et al., 2016).

Among the different models of health behavior change, it is now assumed that there are at least two main stages or phases of behavior change, a *motivational* stage that ends with the formation of a behavioral intention, and a *volitional* stage that ends with successful performance of the behavior; see Gollwitzer (1999), Schwarzer (2008). For the motivational stage there is an on-going debate over the content of the message to address to those targeted. While the proponents of the theory of planned behavior (TPB, Ajzen, 1991) assume that health messages should frame people’s salient beliefs in order to change their behavior, adherents of the self-determination theory (SDT; Ryan and Deci, 2017) suggest that the impact of messages on the behavioral change process may vary according to the nature of goal content (i.e., intrinsic *versus* extrinsic) framed.

Therein, the present study is a theory-based intervention that aimed to examine whether message promoting PA behavior should be framed only to fit the salient beliefs of individuals targeted, as presumed by the TPB (Ajzen, 1991), or whether the goal content framed in the message needs to be considered, as presumed by the SDT (Ryan and Deci, 2017).

The TPB (Ajzen, 1991) assumes that a person’s intention to perform a given behavior, such as PA, is a central determinant of that behavior. Intention is an indicator of how hard people are willing to try, and of how much effort they are planning to exert toward the performance of a behavior (Ajzen, 1991). Intention is determined by three conceptually distinct variables: attitude toward the behavior (i.e., a summary evaluation captured in evaluative dimensions such as good–bad, harmful–beneficial and pleasant–unpleasant), subjective norm (i.e., the perceived social pressure that individuals may feel to perform a given behavior or not) and perceived behavioral control (i.e., the perceived ease or difficulty associated with execution of the behavior in the future) (Ajzen, 1991).

Because attitude, subjective norm, and behavioral control are assumed to be based on corresponding sets of beliefs – namely, behavioral beliefs, normative beliefs, and control beliefs – behavioral interventions have to change such beliefs in order to change intention and eventually behavior (Ajzen, 1991). For this, the theory advises providing persuasive communications targeting modal accessible beliefs (i.e., the most commonly held beliefs in the population). For example, studies (e.g., Hagger et al., 2001) showed that youth-salient behavioral beliefs (i.e., the beliefs that are readily accessible in the memory) toward PA include “to get fit/stay in shape,” “to have fun,” “to improve their skills,” “to make friends,” “ to explore something new,” “to fulfil a fantasy or dream,” “to have a sense of escape or adventure.” As a result, according to TPB, messages should be framed to fit the modal beliefs of the target population. The greater the perceived probability that the behavior will produce such desirable outcomes, the stronger the impact of the beliefs on the attitude and in turn on the intention. In other words, it is assumed that people’s behavioral beliefs or goals are all equally effective for changing their behavior, and that what matters is that the messages be congruent with people’s salient beliefs. This may not be true for the proponents of SDT.

Within the Goal Content Theory (GCT), a sub-theory of SDT, researchers consider the content of the goals people value. They have differentiated *intrinsic goals* from *extrinsic goals* (Deci and Ryan, 2000). *Intrinsic goals* are those focused toward developing one’s personal interests, values and potentials and are inherently satisfying to pursue. In contrast, *extrinsic goals* are primarily characterized by having an “outward” orientation, with one’s pursuit being directed toward external indicators of worth such as wealth, fame and an appealing image (Vansteenkiste et al., 2006). In the PA domain, the goals of skill development, social affiliation, and health management have been characterized as intrinsic goals, and image improvement and social recognition as extrinsic goals (Sebire et al., 2008). Research has revealed that the two goals are differently associated with PA: intrinsic goals are indirectly and positively related to PA, whereas extrinsic goals are indirectly and negatively related to PA *via* self-determined motivation (Gillison et al., 2006; Sebire et al., 2011).

Little research has examined the effect of goal-framing manipulation on PA behavior (Vansteenkiste et al., 2004a,b; Gallagher and Updegraff, 2012; Gillison et al., 2013; study 3). Vansteenkiste et al.’s work examines the effect of intrinsic goal-framing (i.e., exercise could help you attain the goal of physical fitness) *versus* extrinsic goal-framing (i.e., exercise is useful for attaining the goal of appearing physically attractive) conditions on 10th to 12th grades students’ performance and persistence in the tai-bo martial art. In their study Gillison et al. (2013) examined the effect of manipulating adolescents’ goal contents (i.e., health and fitness *versus* looking good to others) on their participation in school physical education. Finally, Gallagher and Updegraff (2012) examined the interaction effect between gain-and loss framed messages, intrinsic and extrinsic goal-framing messages (i.e., well-being *versus* appearance), and the need for cognition on undergraduate students’ PA behavior. Results revealed that the effect of goal-framing messages on PA is inconsistent: Vansteenkiste et al. (2004a) showed that an intrinsic goal-framing message was more efficient than an extrinsic goal-framing message in improving sport performance and persistence, but Gillison et al. (2013) revealed the superiority of extrinsic goal-framing conditions on PA intention, while Gallagher and Updegraff (2012) showed that goal-framing manipulation had no effect on PA participation. This inconsistency may be due to differences in the measure of the dependent variable: there is a gap between intention and the actual PA behavior (Rhodes and de Bruijn, 2013), and between a self-reported and an objective measure of PA (Dale et al., 2002). Thus, to more accurately assess the effect of the goal-framing manipulation on PA duration and intensity, an accelerometer was used in the present study. Another important result from the literature is that intrinsic goal-framing messages did not seem efficient in improving adolescent’s PA participation, in particular when health was targeted. Indeed, health is not a salient goal for healthy young individuals because they do not interpret PA as health behavior (Renner et al., 2007). As a result, knowing whether the goal contents targeted in the messages are all equally effective for changing adolescents’ PA behavior remains an open question. To examine this more fully, the intrinsic goal-framing condition of the present study targeted well-being – a more salient belief for adolescents – rather than health.

Finally, the lack of evidence of the efficacy of framed messages promoting PA on PA behavior shows that intention is a necessary but not a sufficient condition (Rhodes and de Bruijn, 2013). In other words, to improve PA behavior it is not only necessary to increase intentions but also crucial that these intentions do not remain as purely wishful thinking. Planning is therefore a promising strategy for converting intention into action.

Two conceptually distinct forms of planning have been identified in the health domain (Carraro and Gaudreau, 2013; Hagger and Luszczynska, 2014; Hagger et al., 2016). The *action planning* (AP) approach involves specifying multiple cues and complex behavioral responses (Hagger and Luszczynska, 2014). It assumes that cues-to-action should make reference to time-related cues (“when”), the complex external environment (“where”), and the specification of “how” the behavior should be done. AP is generally accompanied by additional coping plans. *Coping planning* (CP) involves the anticipation of barriers or obstacles that could get in the way of the goal striving process, and the generation of alternative behaviors to overcome these difficulties (Sniehotta et al., 2005).

Planned self-regulatory strategies are used in the post-intention phase to bolster intentions to promote recall and enactment of the intended behavior. This action plan makes the goal much more attainable because the mental representation of the anticipated situation becomes highly activated and thus easily accessible (Gollwitzer, 1999). Action initiation becomes swift and efficient and does not require conscious intent, because the direct control of the behavior passes into the environment (Gollwitzer, 1999). In a meta-analysis, Carraro and Gaudreau (2013) revealed a small-to-medium summary (i.e., including the effect of both AP and CP) effect size on PA in experimental studies (φ = 0.24) when comparing all experimental conditions *versus* all controls, and a medium-to-large summary effect (φ = 0.37) when comparing purely planning conditions *versus* neutral controls.

In addition to its effect on the post-intention phase, planning has been shown to be an effective strategy in the pre-intention phase by yielding an implemental mindset. Focusing on information that helps people to achieve the chosen goal, this implemental mindset is associated with higher personal control and probabilities of success; see Achtziger and Gollwitzer (2010), Gollwitzer et al. (2010). In the PA domain, Tessier et al. (2015) compared the effect of planning interventions with two persuasive communications–one framing non-salient beliefs (i.e., health) and another targeting salient beliefs (i.e., fun, affiliation, success, challenge, skills development, and fitness)–on PA behavior, intention, attitude, and perceived behavioral control of low-active adolescents. Results showed a positive effect of a planning intervention on low-active adolescents’ perceived behavioral control and intention to carry out PA, but no effects from the two persuasive communications on PA behavior and TPB variables. In sum, this study highlighted two interesting findings: (1) planning is an effective strategy for increasing intention, and (2) a communication targeting adolescents’ salient beliefs, and close to intrinsic goals (i.e., fun, affiliation, success, challenge, skills development, and fitness), seems inefficient in increasing intention and PA behavior of low-active adolescents.

The purpose of the two present studies was to investigate whether, in a framing message that targets young people’s salient beliefs, the type of goal framed plays a role. In the first study we examined the effect of intrinsic *versus* extrinsic-framing messages on intention and exercise goals. Then, in the second study, we investigated the effects of these two goal-framing conditions combined with planning on the low-active adolescents’ objectively assessed PA behavior, in comparison with a control condition. In this second study, we also examined the mediation effects of TPB variables and the two exercise goals between conditions and PA behavior. In sum, the first study was a pilot study that aimed to develop and test the two goal-framing messages, and the second study was the main trial including a control condition, a planning intervention, an objective measure of PA behavior, and a mediation analysis.

## Study 1

Study 1 consisted in constructing the intrinsic-(i.e., targeting well-being) and extrinsic-(i.e., targeting appearance) framing messages, and testing the effect of these messages on low-active students’ intention, attitude, perceived behavioral control, and exercise goals (i.e., intrinsic and extrinsic). It was hypothesized that the message targeting well-being would increase intrinsic exercise goals but not extrinsic ones, and that the message targeting appearance would increase extrinsic exercise goals, but not intrinsic ones. In addition, it was anticipated that both messages would increase intention and attitude, but not perceived behavioral control, because salient behavioral beliefs, but not salient control beliefs, were targeted in these two messages.

### Materials and methods

#### Participants and procedure

Ten classes of 10th and 11th grades from a high school in a middle-size town in South East France volunteered to be involved in this study. Only the low-active students were included in the intervention. To be considered as a low-active student, the eligibility criterion was to carry out less than 1-h per day of MVPA (World Health Organization, 2010). All the parents were informed and authorized their child to complete the measures. After the study, all students were thanked for their participation and debriefed.

The design was a cluster-randomized trial. Using a random-number table, the study’s main researcher assigned five classes to each of the two conditions of the trial: (a) the extrinsic (EMC) and (b) the intrinsic (IMC) framing-message conditions. Participants were blind to the condition to which their class was assigned. They were told that the study was a survey about PA at adolescence.

The study was conducted in two steps by a researcher during the students’ usual classes. Baseline data were collected 2 weeks before the intervention. The adolescents completed a questionnaire measuring their attitude, perceived behavioral control, intention to be physically active over the next 2 weeks, exercise goals, and past PA behavior. Post-test data collection was conducted immediately after the intervention. Participants completed the same measures as for baseline, except PA. PA was only completed at baseline in order to identify the low-active adolescent (i.e., those who carried out less than 1-h per day of MVPA) within each class. The other students (i.e., more active ones) did not attend the intervention, and did not complete the post-test.

On the basis of the effect size (η^2^ = 0.10) on intention observed in Gillison et al. (2013), and the correlation between repeated measures of intention (*r* = 0.78) reported by Tessier et al. (2015), we calculated using G*Power (Faul et al., 2007) software that 90 participants are needed to reach a power of 0.80 in a design composed of two groups and two times measurements. Among the students who completed baseline measures, 88 were low-active students. All these 88 completed the post-test measures. The sample represented 39 students in the EMC (*M* age = 16.89; 28 females and 11 males) and 49 in the IMC (*M* age = 16.89; 38 females and 11 males).

#### Measures

##### Translation into French

Before the study, each measure was translated into French following the guidelines recommended by Brislin (1980). Each English measure was translated into French using a professional English-French translator. Once done, two native French graduate students who are fluent in both languages then carried out separate back-translations into English. Any discrepancies that emerged between the translators were discussed until a consensus translation was found.

##### Self-reported physical activity

The French adaptation of the International Physical Activity Questionnaire for Adolescents (IPAQ-A, Hagströmer et al., 2008) was used to assess the frequency and intensity of adolescents’ PA behavior over the week prior to baseline (Tessier et al., 2015). This questionnaire covered four domains of PA: (1) school-related PA, including activity during PE classes and breaks, (2) transportation, (3) household, and (4) leisure time, including sport in clubs and unstructured sport activity. Outcome measures were minutes per day reported in walking, moderate and vigorous activity. Time spent in moderate and vigorous activities were summed to obtain an MVPA score for each participant. The IPAQ-A was found to be valid for assessing activities of different intensities and for total PA in healthy European adolescents aged 15–17 years (Hagströmer et al., 2008).

##### Intention

Items drawn from Rhodes and Courneya (2005) were used to measure intention: “Over the next 2 weeks, I intend to do physical activity,” which was anchored by (1) “never” to (7) “every day,” and “I intend to do moderate to vigorous physical activity for at least 7 h/week, over the next 2 weeks,” which was anchored by (1) “definitely no,” to (7) “definitely yes.” Measure of intention reached a satisfactory level of internal consistency at pre-test (α = 0.76) and at post-test (α = 0.80).

##### Perceived behavioral control

Perceived behavioral control was measured by three items drawn from Rhodes and Courneya (2005): “How confident are you that over the next 2 weeks you could exercise at least 5 h/week if you wanted to do so,” which was anchored by (1) “very unconfident,” to (7) “very confident”; “How much personal control do you feel you have over exercising at least 7 h/week in the next 2 weeks,” which was anchored by (1) “very little control,” to (7) “complete control”; and “How much I exercise in the next 2 weeks is completely up to me,” which was anchored by (1) “strongly disagree,” to (7) “strongly agree.” Measure of perceived behavioral attained a satisfactory level of internal consistency at pre-test (α = 0.79) and at post-test (α = 0.83).

##### Attitude

Attitude was measured using responses to one open-ended statement: “For me, exercising 7 h/week over the next 2 weeks would be.” This statement was paired with 5 bipolar seven-point adjective scales (useless–useful, bad–good, harmful–beneficial, unenjoyable–enjoyable, boring–interesting) as previously utilized by Chatzisarantis et al. (2008). Measure of attitude reached a satisfactory level of internal consistency at pre-test (α = 0.81) and at post-test (α = 0.82).

##### Exercise goal content

The Goal Content for Exercise Questionnaire (GCEQ; Sebire et al., 2008) is a 20-item measure that assesses the importance that people place on three intrinsic (health management, skill development, and social affiliation) and two extrinsic (image and social recognition) exercise goals, each indexed by four items. Participants responded to the stem “please indicate to what extent these goals are important for you while exercising” using a seven-point scale ranging from 1 (not at all important) to 7 (extremely important). In the present work, only the items considered as relevant to the message content (i.e., well-being and appearance) were used. Fourteen of the twenty items were identified as relevant: the eight extrinsic items and six of the twelve intrinsic items. Indeed, two items measuring health management (i.e., to increase my resistance to illness and disease; to improve my overall health), one item measuring social affiliation (i.e., to develop close friendships), and three items measuring skill development (i.e., to acquire new exercise skills; to learn and exercise new techniques; to become skilled at a certain exercise or activity) were not meaningfully related to the psychological and physical well-being targeted in the intrinsic goal-framing message. Confirmatory Factor Analysis (CFA) showed good fit of the data to the two-factor structure, after removing two items from the extrinsic dimension that were associated with multiple standardized residuals > ∓ 2.00 [χ^2^(46) = 72.09; *p* < 0.05; χ^2^/df = 1.56; GFI = 0.95; TLI = 0.97; CFI = 0.98; SRMR = 0.06]. The internal consistency for intrinsic (α_*pre–test*_ = 0.88; α_*post–test*_ = 0.90) and extrinsic (α_*pre–test*_ = 0.82; α_*post–test*_ = 0.85) goal content factors reached satisfaction.

#### Framing-messages

With the aim of improving the population’s health by acting on two key determinants - nutrition and PA - the French government has been sponsoring the Program National Nutrition Santé (National Nutrition and Health Program). Adapted from the core information from the Program’s website,^1^ EMC and IMC presentations were created to promote PA. Messages were structured to answer three questions: (1) what (i.e., presentation of the rational of the intervention); (2) why (i.e., development of the core arguments of the message emphasizing the pursuit of intrinsic or extrinsic exercise goals); and (3) how (i.e., proposition of some guidelines to reach these goals in everyday life). The first and third parts of the intervention were identical in the two conditions (to obtain the slides of the framing-messages, please contact the first author).

The EMC and the IMC presentations consisted in delivering a sixteen-slide PowerPoint message. The EMC presentation was entitled “physical activity is good for my appearance,” and the IMC presentation was entitled “physical activity is good for my well-being.” The duration of both interventions was 20 min. Part 1 comprised four slides: two slides were devoted to describing the decrease of PA during adolescence, and two other slides presented guidelines in terms of the intensity and frequency of PA considered good for health: “It is recommended to accumulate at least 1 h or more of MVPA every day of the week. PA can be done in one go or in 10 to 30-min sessions.”

In part 2, nine slides presented the main benefits of PA in terms of appearance and image for EMC, and in terms of well-being for IMC. For example, in the EMC presentation it was said that “doing PA regularly makes you slimmer because your metabolism burns fat,” “doing PA regularly tones the figure, for example running tones up your leg muscles,” “doing PA regularly makes the body appear more dynamic and attractive; people are more interested in and more tolerant toward attractive people.” In contrast, in the IMC presentation it was stated that “doing PA regularly is good for your psychological well-being because of its anxiolytic effect and because it enhances mood, restores mental availability and aids concentration,” “doing PA regularly is good for your physical well-being because it increases your fitness which is related to the feeling of vigor and energy; and also it is strengthens your cardio-vascular system which increases your ability to make sustained effort in your everyday life.”

In part 3, three slides were dedicated to explaining that, “you can reach these recommendations by doing any sports you like (e.g., football, dancing, swimming, and jogging), by setting realistic goals (i.e., progressivity is the key principle), and that every day there are occasions to accumulate short bouts of PA (e.g., going to school on foot or by bike).”

The interventions were delivered by the main researcher at the beginning of the usual lessons. For each condition, the message was standardized and rehearsed several times to ensure the intervention was delivered in the same way in the different classes.

#### Data analysis

Given the nesting nature of the data (i.e., students are nested within classes), intraclass correlations (ICC) were performed. Results revealed that all ICCs were below 5%, meaning that the dependent variables were not influenced by a class-effect. Thus, series of 2 Conditions (EMC *vs.* IMC) × 2 Times (baseline *vs*. post-test) repeated measure ANOVAs were performed in order to compare the effects of each condition on exercise goal contents, intention, attitude, and perceived behavioral control. When variance analyses were significant, we conducted pair-wise mean comparisons using the Newman-Keuls *post hoc* procedure.

### Results

Examination of the differences between the two conditions at baseline indicated no differences for intention [*t*(86) = −0.40, *ns*], for perceived behavioral control [*t*(86) = 0.47, *ns*], for attitude [*t*(86) = 0.15, *ns*], for intrinsic goal content [*t*(86) = 0.97, *ns*], and for extrinsic goal content [*t*(86) = 0.04, *ns*].

For extrinsic goal content, the ANOVA revealed that the Condition main effect was not significant: *F*(1, 86) = 1.08, ns, η^2^ = 0.01; the Time main effect was significant: *F*(1, 86) = 14.32, *p* < 0.001, η^2^ = 0.15; and the interaction effect between Condition × Time was significant: *F*(1, 86) = 4.11, *p* < 0.05, η^2^ = 0.05. Pair-wise comparisons revealed that the score for extrinsic goal content increased in the EMC from baseline to post-test (Ms, 3.21 vs. 4.12, *p* < 0.001), but not in the IMC (Ms, 3.22 vs. 3.49, *ns*).

For intrinsic goal content, the ANOVA revealed a main effect of Condition: *F*(1, 86) = 6.33, *p* < 0.05, η^2^ = 0.07; a main effect of Time: *F*(1, 86) = 16.37, *p* < 0.01, η^2^ = 0.19; and an interaction effect Condition × Time: *F*(1, 86) = 5.99, *p* < 0.05, η^2^ = 0.07. Pair-wise mean comparisons revealed that the score for intrinsic goal content increased in the IMC from baseline to post-test (*Ms*, 4.47 *vs.* 5.35, *p* < 0.001), but not in the EMC (*Ms*, 4.16 *vs.* 4.37, *ns*).

The ANOVA carried out on intention revealed a main effect of Time: *F*(1, 86) = 20.24, *p* < 0.01, η^2^ = 0.24; no main effect of Condition: *F*(1, 86) = 0.43, ns, η^2^ = 0.005; and no interaction effect Condition × Time: *F*(1, 86) = 0.16, ns, η^2^ = 0.002. Pair-wise comparisons revealed that the score for intention increased in the whole sample from baseline (*M* = *3.55*) to post-test (*M*: 3.55 *vs.* 4.26, *p* < 0.01).

The ANOVA carried out on attitude revealed that the Condition main effect was not significant: *F*(1, 86) = 0.07, ns, η^2^ = 0.00; the Time main effect was significant: *F*(1, 86) = 13.22, *p* < 0.001, η^2^ = 0.13; and the interaction effect between Condition × Time was not significant: *F*(1, 86) = 0.37, ns, η^2^ = 0.003. Pair-wise mean comparisons revealed that the score for attitude increased both in the IMC (*Ms*, 5.12 *vs.* 5.55, *p* < 0.05), and in the EMC (*Ms*, 5.16 *vs.* 5.47, *p* < 0.05).

The ANOVA carried out on perceived behavioral control revealed no main effect of Condition: *F*(1, 86) = 0.10, ns, η^2^ = 0.001; no main effect of Time: *F*(1, 86) = 1.96, ns, η^2^ = 0.02; and no interaction effect Condition × Time: *F*(1, 86) = 0.23, ns, η^2^ = 0.002.

### Discussion

In accordance with the hypothesis, students were sensitive to the goal content framed in the messages. In each condition, the goal content corresponding to the goal-framing message increased. Additionally, both intrinsic and extrinsic framing messages were effective in developing low-active adolescents’ intention and attitude. This finding is in agreement with the TPB assumption according to which messages are effective as long as they fit participants’ salient beliefs. In addition, and as expected, both framed messages had no impact on perceived behavioral control because the intervention did not target control beliefs (i.e., the antecedents of perceived behavioral control). Taken together, these results revealed that both goal-framing messages that fit adolescents’ characteristics have the effect to increase their motivation for PA quantitatively (i.e., intention), but the quality of motivation induced by these messages is different, as they are associated with specific exercise goal content.

One potential limitation of this study concerns confounding effects. Parts 1 and 3 of the messages might be active ingredients which overlapped with the effect of the goal-framing messages (i.e., part 2). Indeed, in regard to the taxonomy of behavior change techniques developed by Michie et al. (2009), part 1 of the messages–the presentation of guidelines in terms of the intensity and frequency of PA for promoting health–could be considered as a technique aiming to “provide information about behavior-health link.” In addition, part 3 of the messages–information on the “how” to reach the goals in everyday life–could be regarded as a technique aiming to “provide instruction” (i.e., telling the person how to perform a behavior). In the same vein, one could suspect the existence of a potential Hawthorne effect (McCambridge et al., 2014), that is the tendency for the participants to artificially inflate intention scores at T2 because of their awareness of being studied. One option to overcome these potential confounding effects would be to carry out a control condition. This control group would receive the same intervention as the two experimental conditions, but the core of the message (i.e., part 2) would be a no-goal message. This control condition has been included in study 2.

A second limitation is that the framing effect has not been examined on PA behavior. To strengthen the impact of messages on PA behavior, a planning intervention will be provided in study 2, alongside framing messages. In addition to its influence in the volitional phase, the planning intervention could have an impact in the motivational phase, by increasing the perceived behavioral control as shown by Tessier et al. (2015). As a result, study 2 will extend study 1 by adding a control condition, a planning intervention, and a measure of PA behavior.

## Study 2

Study 2 was carried out to test the effect of intrinsic and extrinsic-framing messages alongside planning on low-active adolescents’ PA. In addition, one central question in the health PA domain is, not only to design effective interventions to promote PA, but also to understand “why” or “why not” a PA intervention worked (Rhodes and Pfaeffli, 2010). To understand this, it is essential to identify the mediators that are needed to change in order that behavior change follows. Thus, the purpose of this second study was twofold. The first aim was to test the effect of intrinsic and extrinsic-framing messages alongside planning on low-active adolescents’ PA, intention, attitude, perceived behavioral control, and exercise goals (intrinsic and extrinsic). The second aim was to examine the mediation effect of these potential mediators within the PA change process. To investigate these two aims, three groups were compared: in one group participants received an intrinsic-framing message and a planning intervention (IMC + P), in a second group participants received an extrinsic-framing message and a planning intervention (EMC + P), and the last group was a control condition (CC) in which participants received a no goal framing message and did not receive a planning intervention. In this study, PA was assessed using an accelerometer during the week after the intervention, and the socio-cognitive mediators were measured at baseline (i.e., 2 weeks before the intervention) and post-test, and the intention was measured again at follow-up (i.e., 2 weeks after the intervention).

It was hypothesized that, compared to CC, adolescents in the two experimental conditions (i.e., IMC + P and EMC + P) would be more physically active, and would report greater intention, more positive attitude, and higher perceived behavioral control. This positive effect of the intervention on intention was expected to be maintained for at least 2 weeks after the intervention. No significant difference was expected between the two experimental conditions concerning the effect of the intervention on TPB variables given that both conditions were elaborated to fit adolescents’ salient behavioral beliefs and control belief. In addition, it was anticipated that from pre-test to post-test, (1) adolescents in the IMC + P would report an increase for intrinsic goals compared to EMC + P and CC; while (2) adolescents in the EMC + P would report an increase for extrinsic goals compared to IMC + P and CC. Finally, while it was expected that both IMC + P and EMC + P would affect PA behavior positively, given the inconsistency of the literature on the effect of intrinsic and extrinsic goal framing messages on PA behavior, no specific hypothesis was made concerning the differentiated effect of these two experimental conditions.

As for aim 2, the effect of conditions was examined using two contrast variables: one contrasting the control condition *versus* the experimental conditions, and the second one contrasting the IMC + P *versus* the EMC + P. It was anticipated that intention and perceived behavioral control measured at time 2 would mediate the effect of the variable “control condition *versus* experimental conditions” on PA behavior, and that intrinsic and extrinsic exercise goals measured at time 2 would mediate the effect of the variable “IMC + P *versus* EMC + P” on PA behavior. All these mediation effects are expected to be partial, because the planning intervention included in the experimental conditions is supposed to have a direct effect on PA behavior.

### Materials and methods

#### Participants and procedure

The sample size was determined on the basis of two formal power analyses (conducted *via* GPower; Faul et al., 2007), one on intention and another on PA behavior. To reach 80% power to detect an effect size of η^2^ = 0.10 of the framing messages on intention (observed in Gillison et al., 2013), and a correlation between repeated measures of intention of *r* = 0.78, (reported by Tessier et al., 2015), the power analysis indicated that 74 participants are needed in a design composed of three groups and two measurement times. The second power analysis, which assumed an effect size of φ = 0.24 for the planning intervention on PA behavior (based on the meta-analysis of Carraro and Gaudreau, 2013), indicated that 171 participants are needed to reach 80% power in a design composed of three groups and one measurement time. Thus, we choose this latter power analysis, which is more conservative than the former, to determine the sample size.

The school administrators of 25 high schools in a middle-size town in South East France were contacted by mail. Three high schools with similar socio-demographic variables volunteered to participate in this study, resulting in the involvement of fifteen classes composed of 193 low-active students.^2^ In multiple measurement occasions, all 193 low-active students were included in the trial. All the parents were informed and authorized their child to complete the measures. After the study, all students were thanked for their participation and debriefed. As for study 1, the design was a cluster-randomized trial. The fifteen classes of 10th and/or 11th grade were randomly assigned to each of the three conditions of the trial: (a) five classes were assigned to EMC + P, (b) five classes were assigned to IMC + P, and (c) five classes were assigned to CC.

The participants were blind to the condition to which their class was assigned, of the participation in the study of other classes from the same school, and no reference was made to the participants about the objective of the study. Instead, they were told that the researchers were carrying out a survey about PA at adolescence.

The study was conducted in three steps by a researcher within the students’ usual PE classes. Baseline data were collected 2 weeks before the intervention. The post-test data collection was conducted immediately after the delivery of the intervention. Finally, the follow-up took place 2 weeks after the intervention.

At baseline, the adolescents completed a questionnaire assessing attitude, perceived behavioral control, intention, exercise goals, and PA behavior. This self-reported measure of PA behavior referred to a normal week and allowed the low-active adolescents–carrying out less than 1-h-per-day of MVPA–in each class to be selected. Just after the intervention (i.e., post-test), the participants completed all baseline measures, except PA. In addition, they received an accelerometer that they had to wear for 1 week. At follow-up the participants completed another measure of intention, in order to test whether the effect of the intervention on intention would be maintained over time.

**FIGURE 1:**
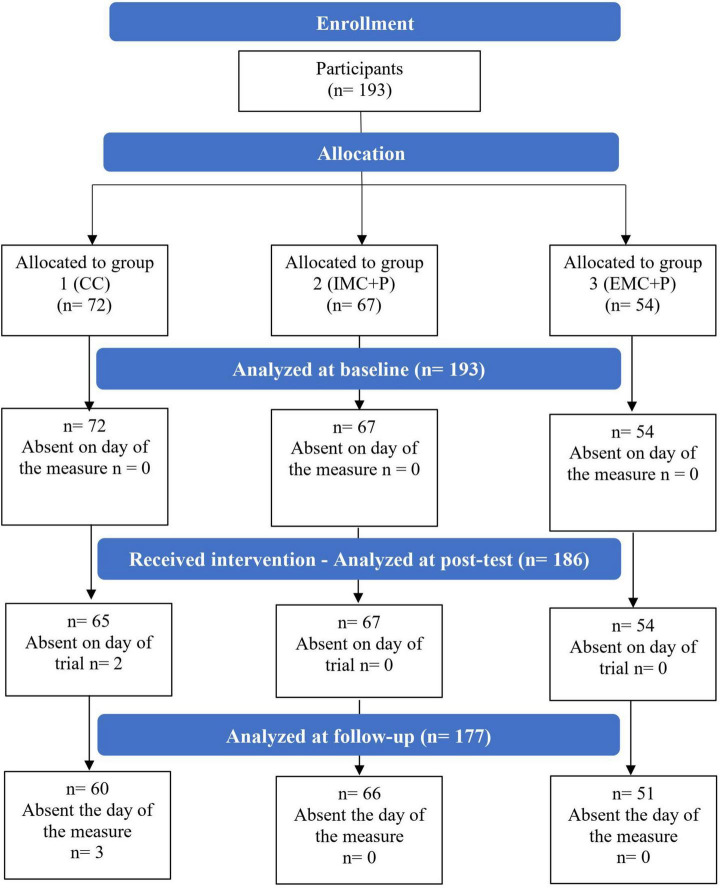
Flow diagram of progress though the trial.

The flow diagram of progress through the trial is depicted in Figure 1. The baseline sample of 15 10th and 11th grade classes represented 193 low-active adolescents (69% female; *M* age: 15.62 years). At post-test, 186 students (69% female; *M* age: 15.71 years) completed the second wave of data collection. The retention rate at post-test 1 was 96%. The 186 persisting students did not differ significantly from the seven students who dropped out at post-test, on the dependent measures (*t*s < 1.5, *ns*). Finally, at follow-up, 177 students (71% female; *M* age: 15.45 years) completed the last wave of data collection. The retention rate at follow-up was 98%. The 177 persisting students did not differ significantly from the nine students who dropped out at follow-up, on intention (*t*s < 1.5, *ns*).

#### Intervention

Interventions in the three conditions were made by a researcher at the beginning of the usual lessons. For each condition, the message was standardized and rehearsed several times to ensure the intervention was delivered in the same way in the different classes. In the two experimental conditions, the intrinsic or extrinsic framing message was delivered first, followed by the planning intervention. The EMC and IMC were the same as in study 1. The planning intervention was based on Gollwitzer and Brandstätter’s (1997) study. A three-slide power point presentation was devoted to informing students on the beneficial impact of planning: “often, it is not easy to organize oneself to successfully reach one’s goals. Many reasons can interfere with your wish to do more. PA planning has been found to facilitate the enactment of the individuals’ desired actions. PA planning is essential for meeting daily recommendations and overcoming potential barriers. For example, if 1 week you have too much homework that does not allow you to be sufficiently active, you may plan to participate in PA during the weekend. “Then, participants were instructed to fill out a week’s timetable. Firstly, they had to write in the timetable slots how long their past PA behaviors lasted in terms of transportation (e.g., walking 10 min to go to school), sport club training/competitions and PE classes (e.g., 2 h football training every Wednesday afternoon, and 1-h PE class each Monday morning and Thursday afternoon), and unstructured leisure time activities (e.g., swimming on Sunday morning for 1 h). Then, if the sum of the usual weekly leisure time PA was less than 7 h, they were invited to plan how to achieve the 7 h of MVPA by adding supplementary PA that they felt able to carry out over the next 2 weeks. Using the *if-then* format, they had to write the type of activity, the day, the time, and the duration of this activity (e.g., If, on Tuesday I get back home at 5pm, I will go for a 30-min run). Finally, the students who planned to do more activities were invited to indicate three possible obstacles that could occur during the pursuit of their goal (i.e., doing at least 7 h of MVPA a week), and three strategies for managing those obstacles (e.g., “If I feel too tired at the end of the day to carry out PA, then I will tell myself that carrying out PA will give me more energy). The duration of the planning intervention was 10 to 15 min. Participants were given a copy of their written plan and were advised to keep this sheet so that it was accessible daily.

The CC consisted in delivering a sixteen-slide PowerPoint message, and the duration of the intervention was 20 min. The structure of the message was similar to those of the EMC and the IMC presentations, except that the seven slides relating to parts 1 and 3 were grouped at the beginning of the message. The CC was entitled “Physical activity and adolescence” (to obtain the slides of this message, please contact the first author). The core component of the message consisted in a nine slide no-goal-framed message that is, a presentation of neutral information that explained the reasons why PA decreases during adolescence. Reasons related to cognitive development (e.g., “during adolescence cognitive skills broaden to virtual objects which increases the tendency to spend more time on the internet and video games leaving less time for PA”), goal conflicts (e.g., “during adolescence the range of interests increases, and adolescent need to make choices between different leisure activities; often PA is sacrificed”), and gender identity (e.g., “adolescents who developed a masculine gender are more likely to do sport, because perseverant stereotype in the sport domain remains: sport is for men.”) were developed. In the CC, the students did not receive the planning intervention.

#### Measures

Measures of *self-reported Physical activity* (i.e., the IPAQ-A), *exercise goal content* (i.e., the 12-item adapted version of the GCEQ), and *TPB* (i.e., intention, attitude, and perceived behavioral control) were the same as in study 1.

##### Direct physical activity measure

Objective PA was measured using the GT3X (Actigraph^®^). The GT3X is a lightweight and compact accelerometer (i.e., 27 grams; dimensions: 3.8 cm × 3.7 cm × 1.8 cm), worn on the waist with an elastic belt. Participants were asked to wear the accelerometer on the right hip during all waking hours (Ward et al., 2005) and remove it only for water activities, bathing or showering. The GT3X accelerometer has shown to be marked by adequate validity, reliability and feasibility when employed in studies of young people (Verloigne et al., 2012). An epoch length of 15 seconds was chosen to capture short durations of high intensity PA (Rowland, 2007). Outcome variables extracted from the accelerometer data were [light PA (LPA); moderate PA (MPA), and vigorous PA (VPA)]. MVPA is defined as the sum of MPA and VPA. Time spent in different PA intensities was determined using age-specific cut-points developed by Freedson et al. (2005). PA intensities were defined as light, moderate, or vigorous for 1.50-2.99, 3.00-5.99 METs^3^, ≥ 6 METs, respectively [i.e., cut- points were between 1,017 and 1,547 counts per minute (cpm) for 3.00 MET at all age groups].

Data were downloaded with ActiLife software. Meterplus version 4.2 was used to screen and clean the data. Consistent with a recent study assessing PA in a representative sample of French children and adolescents (Van Hoye et al., 2014), non-wear time was defined as periods of more than 30 min of consecutive zero counts. Participant data were considered valid and retained for further analysis if data was recorded for a minimum of 8 h per day, on at least three days (i.e., 2 weekdays and 1 weekend day) (Ward et al., 2005). As a result, 49 participants were excluded for insufficient wear time. Data from participants who wore the GT3X for 7 days were used for analysis regarding the number of active days (*N* = 131). The 49 students who were excluded did not differ significantly from the 131 students who wore the GT3X regularly, on the dependent measures (*t*s < 1.5, *ns*).

#### Data analysis

Given the nesting nature of the data (i.e., students are nested within classes), intraclass correlations (ICC) were performed. Results revealed that all ICCs were below 5%, meaning that the dependent variables were not influenced by a class-effect. Thus, to compare the effects of each condition on each variable, series of repeated measures multivariate analysis of variance (MANOVAs) were performed. The required conditions to perform ANOVAs were examined using Kolmogorov–Smirnov and Shapiro-Wilk tests for the normality of the data, and the Levenne test and Cochran, Hartley, Bartlett’s tests for homoscedasticity.

To examine the mechanisms at play, path analyses were conducted with AMOS Version 22.0 (Arbuckle, 2013) using the maximum-likelihood estimation. To test the effects of the three conditions on the potential mediators (i.e., intention, perceived behavioral control, and exercise goals) and PA, we computed two orthogonal contrasts (Judd and McClelland, 2008). One contrast compared the CC with the two experimental conditions (by using −2, 1, and 1, respectively, for CC, EMC + P, and IMC + P), and was labeled “CC vs. EMC + P-IMC + P”; the other contrasted the EMC + P with the IMC + P (by using 0, −1 and 1), and was labeled “EMC + P vs. IMC + P.” “CC vs. EMC + P-IMC + P” was specified as a predictor of intention, attitude, and perceived behavioral control measured at time 2, and PA variables (i.e., MVPA and LPA). “EMC + P vs. IMC + P” was specified as a predictor of intrinsic and extrinsic goals measured at time 2, and PA variables. Based on TCP, attitude and perceived behavioral control measured at time 2 were specified as predictors of intention measured at time 2. The potential mediators, except for attitude which is not directly related to behavior according to TCP, were specified as predictors of the PA variables. In addition, the auto-regressive effective effect (i.e., the effect of the mediator measured at time 1 on itself at time 2) of these potential mediators, and the effect of sex on PA variables were controlled. Two models were examined separately, one with MVPA and one with LPA. To evaluate overall model fit, several indices were used: the chi-square goodness-of-fit statistic, the Tucker–Lewis index (TLI), the Comparative Fit Index (CFI), and the root mean square error of approximation (RMSEA). According to Hu and Bentler (1999), CFI and TLI values above.95 and RMSEA values of less than 0.06 represent a good model fit.

### Results

#### Preliminary analyses

Supplementary Table 1 presents descriptive statistics for the measures; all measures reached satisfactory levels of internal consistency (α > 0.70). Correlations indicated that all TPB measures are positively intercorrelated and positively correlated with goal content. As for PA behaviors, they do not correlate with socio-cognitive variables, except intention.

Examination of the differences between the three conditions at baseline indicated no differences for intention (*p* = 0.07), for perceived behavioral control (*p* = 0.53), and intrinsic goal content (*p* = 0.58), but significant differences for attitude and extrinsic goal content. The participants in the CC had higher attitude scores than those from IMC and EMC (i.e., 5.48 vs. 4.89 and 4.51, *ps* < 0.05), and higher scores for extrinsic exercise goals than those from the EMC (3.7 vs. 2.9, *p* < 0.05). These differences are controlled in the following repeated measures ANOVAs. Possible associations between age and sex with the study’s dependent variables were also tested. Sex was associated with 2 of 5 outcome measures assessed at baseline, with males scoring higher than females on intention, *t*(191) = −3.25, *p* < 0.01 (*Ms*, 4.11 *vs*. 3.28), and perceived behavioral control, *t*(191) = −2.32, *p* < 0.05 (*Ms*, 5.03 *vs*. 4.43). Age was correlated with none of the five student outcomes at baseline. Given these associations, we included sex (females = 0; males = 1) as a covariate (i.e., statistical controls) in the analyses related to these variables.

#### Effects of experimental intervention conditions

For PA behaviors, a MANOVA was performed to compare the three conditions on the PA behaviors. Results showed a significant multivariate effect, *Wilks’ lambda* = 0.41, *F*(4, 254) = 36.01, *p* < 0.001. Follow-up ANOVAs were significant for light PA, *F*(2, 128) = 82.79, *p* < 0.001, η^2^ = 0.56, but not for MVPA, *F*(2, 128) = 0.73, *ns*. More specifically, for light PA, pair-wise mean comparisons (i.e., Newman-Keuls *post hoc* procedure) showed that the scores for EMC + P (*M* = 118.17) and IMC + P (*M* = 122.23) were not different, but significantly higher than the score for CC (*M* = 66.93, *p* < 0.001).

For TPB socio-cognitive variables, only attitude and perceived behavioral control were included in the repeated measures MANOVA, because intention was measured three times. Results showed a significant multivariate interaction effect Time × Condition: *Wilks’ lambda* = 0.91, *F*(4,364) = 4.29, *p* < 0.01. Follow up ANOVAs were significant for both attitude and perceived behavioral control. More specifically, for attitude the Condition main effect was significant: *F*(2, 183) = 3.45, *p* < 0.05, η^2^ = 0.04, the Time main effect was significant: *F*(1, 183) = 16.96, *p* < 0.001, η^2^ = 0.08, and the interaction effect Condition × Time was significant: *F*(2, 183) = 6.68, *p* < 0.01, η^2^ = 0.06. Pair-wise mean comparisons revealed that the score for attitude increased from baseline to post-test in the EMC + P (*Ms*, 4.52 *vs.* 5.25, *p* < 0.001), and in the IMC + P (*Ms*, 4.89 *vs.* 5.58, *p* < 0.01), but not in the CC (*Ms*, 5.48 *vs.* 5.36, *ns*). For perceived behavioral control, Sex was included as a covariate in the repeated measures ANOVA. Results showed a main effect of Time: *F*(1, 180) = 4.99, *p* < 0.05, η^2^ = 0.02, no main effect of the Condition: *F*(2, 180) = 0.37, *ns*, a significant interaction effect Condition × Time: *F*(2, 180) = 7.91, *p* < 0.001, η^2^ = 0.08, and a no interaction effect Condition × Time × Sex: *F*(2, 180) = 1.79, *ns*. Pair-wise mean comparisons revealed that the score of perceived behavioral control increased in the EMC + P from baseline to post-test (*Ms*, 4.46 *vs*. 5.22), but not in the IMC + P (*Ms*, 4.56 *vs*. 4.74), and in the CC (*Ms*, 4.73 *vs*. 4.46). As for intention, Sex was included as a covariate in the repeated measures ANOVA. Results showed a non-significant main effect of the Condition: *F*(2, 171) = 0.13, *ns*; a significant main effect of Time: *F*(2, 342) = 39.66, *p* < 0.001, η^2^ = 0.19; a significant interaction effect Time × Condition: *F*(4, 342) = 6.56, *p* < 0.001, η^2^ = 0.07; and a significant interaction effect Time × Condition × Sex: *F*(4, 342) = 2.59, *p* < 0.05, η^2^ = 0.02. Pair-wise mean comparisons revealed that the score for intention in the EMC + P and in the IMC + P increased from baseline to post-test and decreased from post-test to follow-up (for EMC *Ms*, 3.39 *vs*. 5.07 *vs*. 4.38; for IMC *Ms*, 3.32 *vs*. 4.86 *vs*. 4.07). In the CC the scores for intention leveled off (*Ms*, 3.83 vs. 4.23 *vs*. 4.20). The pattern of results is identical for both males and females, but the scores for intentions are higher for males than for females.

For students’ goal content, a repeated measures MANOVA was performed to compare pre-test to post-test. Results showed a significant multivariate interaction effect Time × Condition: *Wilks’ lambda* = 0.85, *F*(4,358) = 7.39, *p* < 0.001. Follow-up ANOVAs were significant for both intrinsic and extrinsic goals. More specifically, results of repeated measures ANOVA on extrinsic goal showed a main effect of Condition: *F*(2, 180) = 1.22, *ns*; a main effect of Time: *F*(1, 180) = 30.60, *p* < 0.001, η^2^ = 0.15; and an interaction effect Condition × Time: *F*(2, 180) = 10.32, *p* < 0.01, η^2^ = 0.10. Pair-wise mean comparisons (i.e., Newman-Keuls *post hoc* procedure) revealed that the score for extrinsic goal increased in the EMC + P from baseline to post-test (*Ms*, 2.96 *vs.* 3.92, *p* < 0.001), but not in the IMC + P (*Ms*, 3.35 *vs.* 3.53, *ns*), and in the CC (*Ms*, 3.69 *vs.* 3.86, *ns*). As for the intrinsic exercise goal, results of repeated measures ANOVA revealed that the Condition main effect was not significant: *F*(2, 180) = 1.19, *ns*; the Time main effect was significant: *F*(1, 180) = 22.05, *p* < 0.001, η^2^ = 0.11; and the interaction effect between Condition × Time was significant: *F*(2, 180) = 3.37, *p* < 0.05, η^2^ = 0.04. Pair-wise mean comparisons revealed that the score for intrinsic goal increased in the IMC + P from baseline to post-test (*Ms*, 4.91 *vs.* 5.62, *p* < 0.001), but not in the EMC + P (*Ms*, 4.85 *vs.* 5.07, *ns*), and in the CC (*Ms*, 5.04 *vs.* 5.32, *ns*).

#### Mediational analysis

**FIGURE 2:**
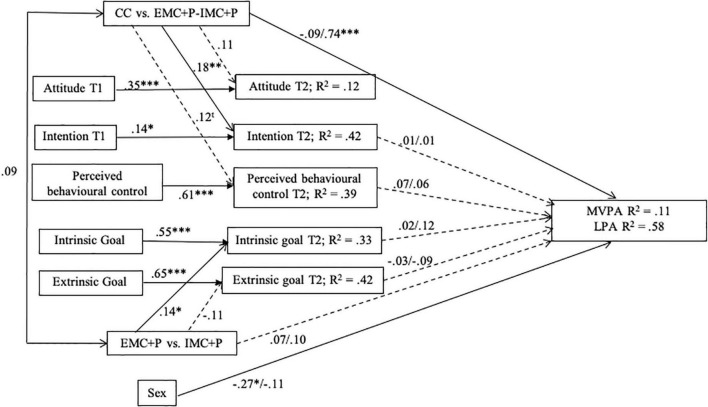
Mediational models. When two indices are noted on the path analysis, the first number corresponds to MVPA, and the second one to light physical activity. Sex is coded as 1 = male and 2 = female.

The models specified yielded a satisfactory fit across indices. For MVPA, χ^2^(39) = 69.69, *p* = 0.004; TLI = 0.94; CFI = 0.96; RMSEA = 0.06 [0.02, 0.09]; and for LPA, χ^2^(39) = 66.72, *p* = 0.004; TLI = 0.94; CFI = 0.96; RMSEA = 0.06 [0.02, 0.09]. As shown in Figure 2, compared to CC, the experimental conditions were positively related to intention at time 2 (ß = 0.18, *p* < 0.01), to LPA (ß = 0.74, *p* < 0.001) but not to MVPA (ß = −0.09, *ns*), and was tendentially associated to perceived behavioral control at time 2 (ß = 0.12, *p* = 0.07). Bootstrap analyses showed that the indirect effects between “CC vs. EMC + P-IMC + P” and LPA/MVPA were not significant through intention at time 2 [−0.09, CI 95%: (−0.97, 0.63); *p* = 0.43, and −0.11, CI 95%: (−0.84, 0.58); *p* = 0.76], and though perceived behavioral control at time 2 [0.21, CI 95%: (−0.20, 0.86); *p* = 0.84, and 0.06, CI 95%: (−0.37, 0.58); *p* = 0.73]. In addition, compared to EMC + P, IMC + P was positively associated with intrinsic goal at time 2 (ß = 0.14, *p* < 0.05), and tendentially related to extrinsic goal at time 2 (ß = −0.12, *p* = 0.08). The experimental conditions were not significantly related LPA (ß = 0.07, *ns*), nor was MVPA (ß = 0.10, *ns*). Finally, none of the socio-cognitive variables measured at time 2 were related to PA behaviors. Bootstrap analyses showed that the indirect effects between “EMC + P vs. IMC + P” and LPA/MVPA were not significant through intrinsic goal at time 2 [0.03, CI 95%: (−0.92, 1.41); *p* = 0.71, and.15, CI 95%: (−0.33, 0.87); *p* = 0.28], and though extrinsic goal at time 2 [−0.11, CI 95%: (−1.71, 1.05); *p* = 0.91, and −0.16, CI 95%: (−0.94, 0.34); *p* = 0.28]. Overall, the models explained 58% of the variance of LPA, and 11% of the variance of MVPA.

### Discussion

The purpose of this second study was to test the effect of intrinsic- and extrinsic-goal framing messages alongside planning on low-active adolescents’ PA, and to investigate the contribution of the potential mediators: intention, attitude, perceived behavioral control, and intrinsic and extrinsic exercise goals.

#### Effect of the intervention on physical activity variables

A noteworthy result is that, compared to low-active adolescents in CC, those in EMC + P and IMC + P did not practice more MVPA, but carried out more LPA. This result is in line with the literature reviews showing the efficacy of the behavior change techniques used in this study on PA behavior, that “provide information on consequences of behavior to the individual” framing intrinsic or extrinsic exercise goal content, and “action and coping planning” (Michie et al., 2011; Carraro and Gaudreau, 2013; Dusseldorp et al., 2014). Additionally, these findings obtained using an accelerometer to measure PA are interesting given that most previous studies used an indirect (i.e., self-reported) measure of PA. Indeed, it is now well-established that indirect measures tend to overestimate the score for PA (Troiano et al., 2008), hence the accelerometer is a more conservative measure of PA than indirect measures. Thus, despite the lack of effect on MVPA, the significant effect obtained on LPA is promising. The lack of effect on MVPA may also be due to a dose-response effect. Indeed, the intervention was short (i.e., about half an hour in duration), and delivered only once. Literature shows that the effect of the messages is enhanced by more frequent doses of information see Latimer et al. (2010).

The lack of difference between IMC + P and EMC + P on PA variables is also interesting. Although the results on intrinsic and extrinsic goals confirm that both experimental conditions are different in nature, they had the same effect on PA behaviors. As the planning intervention was the same in the two experimental conditions, it is possible that either, IMC and EMC yielded an identical significant effect on LPA, or that IMC and EMC had no significant effect on PA, and thus the effect observed was only due to the planning intervention. This is an important limitation of the present study. The lack of a factorial research design – which would enable the identify the main effect of each framing message and of the planning intervention as well as the interaction effects among these factors – do not allow to know whether the framing messages influenced, or not, on the PA behavior. Consequently, the results obtained with these combined experimental conditions do not shed new light on the specific effect of extrinsic and extrinsic goal framing messages on PA behavior.

#### Effect of the intervention on the potential mediators

As in study 1, results showed that in the IMC + P the intrinsic exercise goals increased from pre-test to post-test, and in the EMC + P the extrinsic exercise goals increased over time. In the CC neither intrinsic nor extrinsic exercise goals changed over time. While this result tends to accredit SDT over TPB by showing that students are sensitive to the exercise goal contents framed in the messages, this difference between the conditions seems negligible nevertheless, as no consequence on PA behavior was observed.

In addition, results revealed that both experimental conditions yielded an increase in attitude and intention, but not the CC. These findings confirm the real positive impact of goal framing messages on the variables in the pre-intention phase and, at the same time, the lack of effect of other potential behavioral change techniques included in parts 1 and 3 of the framed messages. Contrary to the limitation evoked for study 1, the information included in parts 1 and 3 of the framing-messages were not active ingredients in the behavioral change process.

In addition, results showed that compared to IMC + P and CC, EMC + P produced an increase of perceived behavioral control. This finding partially confirms the hypothesis. In line with the study of Tessier et al. (2015), it was expected that planning would increase perceived behavioral control in both experimental conditions. In the IMC + P, the increase was not enough to be significant. This may be due to the fact that the baseline level of perceived behavioral control was high in both groups (i.e., > 4 of 7), but slightly more in the IMC + P than in the EMC + P.

Another interesting finding from study 2 is that the framing effect on intention decreased rapidly. Indeed, while in the two experimental conditions follow-up scores of intentions – taken 2 weeks after the intervention – were significantly higher than the pre-test scores, they significantly decreased from post-test to follow-up. Thus, it can be assumed that the positive effect of this brief intervention was short-lived. However, it must be noted that the frame of this study was focused on the initiation phase, and not on maintenance. The maintenance phase is a complex issue (i.e., PA volume is volatile), that requires the use of specific intervention strategies (e.g., follow-up prompts such as maintaining long-term contact with the participants), and specific methods to evaluate long-term effect of intervention (12 months or longer) (Fjeldsoe et al., 2011).

#### Mediational analysis

Path analyses revealed three main findings (see Figure 2). First, “CC vs. EMC + P-IMC + P” was significantly related to LPA. This result confirms the hypothesis and means that providing information on intrinsic or extrinsic consequences of PA to the individual, alongside action and coping planning, has a greater positive effect on LPA than only informing low-active adolescents of guidelines in terms of PA intensity and frequency (i.e., control condition). Second, “EMC + P vs. IMC + P” was not related to PA behaviors. This means that intrinsic goal framing messages had no added-value compared to extrinsic goal framing messages in terms of PA in the short term. This finding tends to confirm the results of Gallagher and Updegraff (2012) who showed no effect of goal-framing manipulation on PA participation. Finally, results showed that none of the potential mediators was significantly related to either MVPA or LPA. This finding invalidates the hypothesis, showing that the effect of the intervention on LPA is not mediated by TCP variables nor intrinsic and extrinsic exercise goals.

## General discussion

The purpose of the two present studies was to investigate whether, when framing messages that target young people’s salient beliefs, the type of goal that was framed is important in promoting PA among low-active adolescents.’ Results showed that both experimental conditions had similar effects on intentions (studies 1 and 2) and on PA behavior (study 2). Thus, while low-active adolescents were sensitive to the message content – as the change in intrinsic or extrinsic goals corresponded to the goal content framed – the intrinsic goal framing message did not bring any added-value to the PA behavior change process compared to the extrinsic goal framing message.

Another finding of this research was to reveal that an intervention combining a message providing information on consequences of PA alongside planning could contribute effectively to promoting PA behavior in low-active adolescents. This finding extends earlier work, in particular that in Tessier et al. (2015) that showed that a combination of a persuasive communication based on adolescents’ salient beliefs and a planning intervention did not increase self-reported PA behavior among low-active adolescents. However, the present study failed to explain the mechanisms at play. Indeed, TCP variables and intrinsic and extrinsic exercise goals did not mediate the effect of the intervention on PA behavior. Other potential mediators warrant investigation, in particular in the volitional phase.

Finally, the present study confirms previous findings (Tessier et al., 2015), emphasizing the crucial role played by action and coping planning in the motivational phase by improving perceived behavioral control, as well as in the volitional phase by increasing PA behavior.

### Limitations and future research

The current set of studies is not without limitations. First, the design does not allow to disentangle the effect of the framing messages and the effect of the planning intervention. To examine separately the main effect of these two interventions, the use of a factorial research design including a fourth condition in which participants would assist to the planning intervention only would be warranted. In addition, to examine the main effect of the framing messages in the IMC + P and the EMC + P, the adding of another measure of the TPB variables and the PA behavior after the delivering of the framing messages would be also warranted. This would imply to implement the planning intervention 1 week apart in these two conditions. Also, to examine the effect of the planning intervention more thoroughly, the inclusion of a self-reported measure of planning at follow-up in all the groups would be necessary. Second, delivering the intervention only once is probably insufficient to increase the MVPA of low-active adolescents. In the future, it would be warranted to have more repetitions of the intervention in order to impact MVPA. Finally, the effects of the intervention at follow-up were examined only at very short term (i.e., within 2 weeks after the intervention) using intention measures. A more useful follow-up measure of this brief intervention focused on PA initiation would have been needed to assess PA behavior two or 3 months after the intervention.

### Conclusion

Low-active adolescents are a population to consider in order preventing future health issues. This study is innovative because it combined two efficient behavioral techniques in a natural setting, and used a direct measure (i.e., accelerometer) to assess the effects of the intervention on PA. The results demonstrated that “providing information on consequences” alongside planning is an effective low-cost intervention in order to increase the LPA of low-active adolescents. In the future, given the lack of interventions to promote PA in low-active adolescents, there is a need to replicate this intervention and to test other innovative interventions to increase PA among this vulnerable population.

## Data availability statement

Publicly available datasets were analyzed in this study. This data can be found here: https://osf.io/vytqp/.

## Ethics statement

Ethical review and approval was not required for the study on human participants in accordance with the local legislation and institutional requirements. Written informed consent to participate in this study was provided by the participants’ legal guardian/next of kin.

## Author contributions

DT developed the study concept, analyzed, interpreted the data, and drafted the manuscript. DT, VN, and PS contributed to the study conception and design. VN contributed to data acquisition. VN and PS provided critical revisions. All authors approved the final version of this manuscript for publishing.

## Footnotes

1
http://www.mangerbouger.fr

2 As for the pilot study, to be considered as a low-active student, the eligibility criterion was to carry out less than 1-h per day of MVPA (World Health Organization, 2010).

3 The Metabolic Equivalent of Task (MET), or simply metabolic equivalent, is a physiological measure expressing the energy cost of physical activities.

## References

[B1] Achtziger A. Gollwitzer P. M. (2010). “Motivation and volition in the course of action,” in *Motivation and action*, 2nd Edn, eds Heckhausen J. Heckhausen H. (New York, NY: Cambridge University Press), 275–299.

[B2] Ajzen I. (1991). The theory of planned behavior. *Organ. Behav. Hum. Decis. Process.* 50 179–211. 10.1016/0749-5978(91)90020-T

[B3] Arbuckle J. L. (2013). *Amos 22.0 User’s Guide.* Chicago: SPSS.

[B4] Biddle S. J. H. Asare M. (2011). Physical activity and mental health in children and adolescents: A review of reviews. *Br. J. Sports Med.* 45 886–895. 10.1136/bjsports-2011-090185 21807669

[B5] Brislin R. (1980). “Translation and content analysis of oral and written material,” in *Handbook of cross-cultural psychology*, Vol. 2 eds Triandis H. C. Berry J. W. (Boston Allyn & Bacon), 389–444. 10.3390/healthcare6030093

[B6] Carraro N. Gaudreau P. (2013). Spontaneous and experimentally induced action planning and coping planning for physical activity: A meta-analysis. *Psychol. Sport Exerc.* 14 228–248. 10.1016/j.psychsport.2012.10.004

[B7] Chatzisarantis N. Hagger M. Brickell T. (2008). Using the construct of perceived autonomy support to understand social influence within the theory of planned behavior. *Psychol. Sport Exerc.* 9 27–44. 10.1016/j.psychsport.2006.12.003

[B8] Dale D. Welk G. J. Matthews C. E. (2002). “Methods for assessing physical activity and challenges for research,” in *Physical activity assessments for health-related research*, ed. Welk G. J. (Champaign, IL: Human Kinetics), 19–34.

[B9] Deci E. L. Ryan R. M. (2000). The “what” and “why” of goal pursuits: Human needs and the self-determination of behaviour. *Psychol. Inq.* 11 227–268. 10.1207/S15327965PLI1104_01

[B10] Dusseldorp E. Van Genugten L. Van Buuren S. Verheijden M. Van Empelen P. (2014). Combinations of techniques that effectively change health behavior: Evidence from Meta-CART analysis. *Health Psychol.* 33 1530–1540. 10.1037/hea0000018 24274802

[B11] Faul F. Erdfelder E. Lang A.-G. Buchner A. (2007). GPower 3: A flexible statistical power analysis program for the social, behavioral, and biomedical sciences. *Behav. Res. Methods* 39 175–191. 10.3758/BF03193146 17695343

[B12] Fjeldsoe B. Neuhaus M. Winkler E. Eakin E. (2011). Systematic review of maintenance of behavior change following physical activity and dietary interventions. *Health Psychol.* 30 99–109. 10.1037/a0021974 21299298

[B13] Freedson P. Pober D. Janz K. F. (2005). Calibration of accelerometer output for children. *Med. Sci. Sports Exerc.* 37 S523–S530. 10.1249/01.mss.0000185658.28284.ba 16294115

[B14] Gallagher K. M. Updegraff J. A. (2012). Health message framing effects on attitudes, intentions, and behavior: A meta-analytic review. *Anna. Behav. Med.* 43 101–116. 10.1007/s12160-011-9308-7 21993844

[B15] Gillison F. B. Standage M. Skevington S. M. (2006). Relationships among adolescents’ weight perceptions, exercise goals, exercise motivation, quality of life and leisure-time exercise behaviour: A self-determination theory approach. *Health Educ. Res.* 21 836–847. 10.1093/her/cyl139 17101718

[B16] Gillison F. B. Standage M. Skevington S. M. (2013). Effect of the manipulating goal content and autonomy support climate and outcomes of a PE fitness class. *Psychol. Sport Exerc.* 14 342–352.

[B17] Gollwitzer P. M. (1999). Implementation intentions: Strong effects of simple plans. *Am. Psychol.* 54 493–503. 10.1037/0003-066X.54.7.493

[B18] Gollwitzer P. M. Brandstätter V. (1997). Implementation intentions and effective goal pursuit. *J. Pers. Soc. Psychol.* 73 186–199. 10.1037/0022-3514.73.1.186 11708569

[B19] Gollwitzer P. M. Wieber F. Meyers A. L. McCrea S. M. (2010). “How to maximize implementation intention effects,” in *Then a miracle occurs: Focusing on behavior in social psychological theory and research*, eds Agnew C. R. Carlston D. E. Graziano W. G. Kelly J. R. (New York: Oxford Press), 137–161. 10.1093/acprof:oso/9780195377798.003.0008

[B20] Gourlan M. Bernard P. Bortolin C. Romain A. J. Lareyre O. Carayol M. (2016). Efficacy of theory-based interventions to promote physical activity. A meta-analysis of randomised controlled trials. *Health Psychol. Behav.* 10 50–66. 10.1080/17437199.2014.981777 25402606

[B21] Hagger M. S. Luszczynska A. (2014). Implementation intention and action planning Interventions in health contexts: State of the research and proposals for the way forward. *Appl. Psychol. Health Well Being* 6 1–47. 10.1111/aphw.12017 24591064

[B22] Hagger M. S. Chatzisarantis N. Biddle S. J. (2001). The influence of self-efficacy and past behaviour on the physical activity intentions of young people. *J. Sports Sci.* 19 711–725. 10.1080/02640410152475847 11522147

[B23] Hagger M. S. Luszczynska A. de Wit J. Benyamini Y. Burkert S. Chamberland P.-E. (2016). Implementation intention and planning interventions in health psychology: Recommendations from the Synergy expert group for research and practice. *Psychol. Health* 31 814–839. 10.1080/08870446.2016.114671 26892502

[B24] Hagströmer M. Bergman P. Bourdeaudhuij I. Ortega F. B. Ruiz J. R. Manios Y. (2008). Concurrent validity of a modified version of the International Physical activity Questionnaire (IPAQ-A) in European adolescents: The HELENA study. *Intl. J. Obes.* 32 (Suppl. 5) S42–S48. 10.1038/ijo.2008.182 19011653

[B25] Hallal P. C. Andersen L. B. Bull F. C. Guthold R. Haskell W. Ekelund U. (2012). Global physical activity levels: Surveillance progress, pitfalls, and prospects. *Lancet (London, England)* 380 247–257. 10.1016/S0140-6736(12)60646-1 22818937

[B26] Hallal P. C. Victoria C. G. Azevedo M. R. Wells J. C. K. (2006). Adolescent physical activity and health: A systematic review. *Sport Med.* 36 1019–1030. 10.2165/00007256-200636120-00003 17123326

[B27] Hu L. Bentler P. M. (1999). Cutoff criteria for fit indexes in covariance structure analysis: Conventional criteria versus new alternatives. *Struct. Equ. Model.* 6 1–55. 10.1080/10705519909540118

[B28] Inchley J. Currie D. Young T. Samdal O. Torsheim T. Augustson L. (eds) (2016). *Growing up unequal: Gender and socioeconomic differences in young people’s health and well-being”, Health Behavior in School-aged Children (HBSC) Study: International Report from the 2013/2014 Survey.* Copenhagen: WHO Regional Office for Europe.

[B29] Judd C. M. McClelland G. H. (2008). *Data analysis: A model comparison approach*, 2nd Edn. San Diego, CA: Harcourt Brace Jovanovich.

[B30] Latimer A. E. Brawley L. R. Bassett R. L. (2010). A systematic review of three approaches for constructing physical activity messages: What messages work and what improvements are needed? *Int. J. Behav. Nutr. Phys. Act.* 7 1–36. 10.1186/1479-5868-7-36 20459779 PMC2885311

[B31] McCambridge J. Witton J. Elbourne D. R. (2014). Systematic review of the Hawthorne effect: New concepts are needed to study research participation effects. *J. Clin. Epidemiol.* 67 267–277. 10.1016/j.jclinepi.2013.08.015 24275499 PMC3969247

[B32] Michie S. Abraham C. Whittington C. McAteer J. Gupta S. (2009). Effective techniques in healthy eating and physical activity interventions: A meta-regression. *Health Psychol.* 28 690–701. 10.1037/a0016136 19916637

[B33] Michie S. van Stralen M. M. West R. (2011). The behaviour change wheel: A new method for characterising and designing behaviour change interventions. *Implement. Sci.* 6:42. 10.1186/1748-5908-6-42 21513547 PMC3096582

[B34] Renner B. Spivak Y. Kwon S. Schwarzer R. (2007). Does age make a difference? Predicting physical activity of South Koreans. *Psychol. Aging* 22 482–493. 10.1037/0882-7974.22.3.482 17874949

[B35] Rhodes R. E. Courneya K. S. (2005). Threshold assessment of attitude, subjective norm, and perceived behavioral control for predicting exercise intention and behavior. *Psychol. Sport Exerc.* 6 349–361. 10.1016/j.psychsport.2004.04.002

[B36] Rhodes R. E. de Bruijn G. J. (2013). How big is the physical activity intention-behaviour gap? A meta- analysis using the action control framework. *Br. J. Health Psychol.* 18 296–309. 10.1111/bjhp.12032 23480428

[B37] Rhodes R. E. Pfaeffli L. A. (2010). Mediators of physical activity behaviour change among adult non-clinical populations: A review update. *Intl. J. Behav. Nutr. Phys. Act.* 7:37. 10.1186/1479-5868-7-37 20459781 PMC2876989

[B38] Rowland A. V. (2007). Accelerometers assessment of physical activity in children: An update. *Pediatr. Exerc. Sci.* 19 252–266. 10.1123/pes.19.3.252 18019585

[B39] Ryan R. M. Deci E. L. (eds) (2017). *Self-determination theory: Basic psychological needs in motivation, development, and wellness.* New York, NY: Guilford Press. 10.1521/978.14625/28806

[B40] Schwarzer R. (2008). Models of health behaviour change: Intention as mediator or stage as moderator? *Psychol. Health* 23 259–263. 10.1080/08870440801889476 25160477

[B41] Sebire S. Standage M. Vansteenkiste M. (2008). Development and validation of the goal content for exercise questionnaire. *J. Sport Exerc. Psychol.* 30 353–377. 10.1123/jsep.30.4.353 18723897

[B42] Sebire S. Standage M. Vansteenkiste M. (2011). Predicting objectively assessed physical activity from the content and regulation of exercise goals: Evidence for a mediational model. *J. Sport Exerc. Psychol.* 33 175–197. 10.1123/jsep.33.2.175 21558579

[B43] Sniehotta F. F. Schwarzer R. Scholz U. Schuz B. (2005). Action planning and coping planning for long term lifestyle change: Theory and assessment. *Eur. J. Soc. Psychol.* 35 565–576. 10.11124/jbisrir-2015-1698 26455612

[B44] Tessier D. Sarrazin P. Nicaise V. Dupont J.-P. (2015). The effects of persuasive communication and planning on intentions to be more physically active and on physical activity behaviour among low active adolescents. *Psychol. Health* 30 583–604. 10.1080/08870446.2014.996564 25493545

[B45] Troiano R. P. Berrigan D. Dodd K. W. Mâsse L. C. Tilert T. McDowell M. (2008). Physical activity in the United States measured by accelerometer. *Med. Sci. Sports Exerc.* 40 181–188. 10.1249/mss.0b013e31815a51b3 18091006

[B46] Van Hoye A. Nicaise V. Sarrazin P. (2014). Self-reported and objective physical activity measurement by active youth. *Sci. Sports* 29 78–87. 10.1016/j.scispo.2013.01.010

[B47] Vansteenkiste M. Lens W. Deci E. L. (2006). Intrinsic versus extrinsic goal-contents in self-determination theory: Another look at the quality of academic. *Educ. Psychol.* 41 19–31. 10.1207/s15326985ep4101_4

[B48] Vansteenkiste M. Simons J. Lens W. Sheldon K. M. Deci E. L. (2004a). Motivating learning, performance and persistence: The synergestic effects of intrinsic goal contents and autonomy-supportive contexts. *J. Pers. Soc. Psychol.* 87 246–260. 10.1037/0022-3514.87.2.246 15301630

[B49] Vansteenkiste M. Simons J. Soenens B. Lens W. (2004b). How to become a persevering exerciser? Providing a clear, future intrinsic goal in an autonomy-supportive manner. *J. Sport Exerc. Psychol.* 26 232–249. 10.1123/jsep.26.2.232

[B50] Verloigne M. Van Lippevelde W. Maes L. Yildirim M. Chinapaw M. Manios Y. (2012). Levels of physical activity and sedentary time among 10- to 12-year-old boys and girls across 5 European countries using accelerometers: An observational study within the ENERGY-project. *Intl. J. Behav. Nutr. Phys. Act.* 9 1–8. 10.1186/1479-5868-9-34 22462550 PMC3359200

[B51] Ward D. S. Evenson K. R. Vaughn A. Rodgers A. B. Troiano R. P. (2005). Accelerometer use in physical activity: Best practices and research recommendations. *Med. Sci. Sports Exerc.* 37 S582–S588. 10.1249/01.mss.0000185292.71933.91 16294121

[B52] World Health Organization (2009). “Global Health Risks. mortality and burden of diseases attributable to selected major risks,” in *Global recommendations on physical activity for health*, ed. Organisation W. H. (Geneva: World Health Organization).

[B53] World Health Organization (2010). *Global recommendations on physical activity for health. World Health Organization.* Available online at: http://whqlibdoc.who.int/publications/2010/9789241599979_eng.pdf 26180873

[B54] World Health Organization (2011). *Global school-based student health survey.* Geneva: World Health Organization.

