# Supplementary material for: The effects of a cluster-randomized control trial manipulating exercise goal content and planning on physical activity among low-active adolescents
Source: Front Psychol. 2022 Sep 14;13:950107. doi: 10.3389/fpsyg.2022.950107 (PMC9516308; doi:10.3389/fpsyg.2022.950107)
Supplement: Supplementary file 1 [file Table_1.docx]

Supplementary Table 1.  *Descriptive statistics and correlations*

|  | 1  Age | 2  Sex | 3  INT | 4  ATT | 5  PBC | 6  IGC | 7  EGC | 8  INT | 9  ATT | 10  PBC | 11  IGC | 12  EGC | 13  LPA | 14  MVPA | 15  INT |
| --- | --- | --- | --- | --- | --- | --- | --- | --- | --- | --- | --- | --- | --- | --- | --- |
| 1 | - |  |  |  |  |  |  |  |  |  |  |  |  |  |  |
| 2 | -.03 | - |  |  |  |  |  |  |  |  |  |  |  |  |  |
| Baseline | | | | | | | | | | | | | | |  |
| 3 | -.15 | -.13 | .83 |  |  |  |  |  |  |  |  |  |  |  |  |
| 4 | -.04 | -.04 | .49*** | .84 |  |  |  |  |  |  |  |  |  |  |  |
| 5 | -.01 | -.07 | .47*** | .51*** | .86 |  |  |  |  |  |  |  |  |  |  |
| 6 | -.12 | -.00 | .44** | .48*** | .25* | .83 |  |  |  |  |  |  |  |  |  |
| 7 | -.06 | -.09 | .28* | .30** | .07 | .48** | .85 |  |  |  |  |  |  |  |  |
| Posttest | | | | | | | | | | | | | | |  |
| 8 | -.16 | -.13 | .44*** | .36** | .38** | .44** | .23* | .78 |  |  |  |  |  |  |  |
| 9 | -.21* | -.14 | .29* | .43*** | .30* | .32* | .22* | .48** | .87 |  |  |  |  |  |  |
| 10 | -.24* | -.16 | .48*** | .47*** | .65** | .37** | .21* | .67*** | .51*** | .88 |  |  |  |  |  |
| 11 | -.19* | .02 | .19* | .34** | .21* | .55*** | .38** | .29* | .28* | .28* | .84 |  |  |  |  |
| 12 | -.14 | -.08 | .23* | .23* | .07 | .41** | .68*** | .24* | .25* | .29* | .39** | .87 |  |  |  |
| 13 | -.29* | -.06 | -.04 | -.14 | .10 | -.05 | -.18* | .21* | .13 | .16 | .03 | -.05 |  |  |  |
| 14 | .17 | -.24* | .01 | .04 | .06 | -.15 | -.04 | -.05 | -.15 | -.01 | -.09 | -.13 | .10 | - |  |
| Follow-up | | | | | | | | | | | | | | |  |
| 15 | -.22* | -.09 | .58*** | .41** | .42** | .43** | .20* | .64*** | .44** | .60*** | .33** | .28* | .06 | .10 | .80 |
| M | 15.62 | - | 3.52 | 5.01 | 4.61 | 4.95 | 3.37 | 4.77 | 5.41 | 4.78 | 5.35 | 3.77 | 101.27 | 34.58 | 4.22 |
| SD | 0.91 | - | 1.68 | 1.49 | 1.70 | 1.31 | 1.50 | 1.54 | 1.21 | 1.42 | 1.12 | 1.48 | 34.41 | 16.61 | 1.57 |
| Median | 15.7 | - | 3.50 | 5.20 | 4.67 | 5.25 | 3.20 | 5.00 | 5.80 | 4.67 | 5.30 | 3.80 | 105 | 34 | 4.00 |
| Skewness | -0.39 | - | 0.51 | -0.87 | -0.39 | -0.49 | 0.27 | -0.28 | -0.99 | -0.23 | -0.77 | -0.005 | 0.52 | 0.26 | -0.10 |
| Kurtosis | 1.04 | - | -0.58 | 0.35 | -0.82 | -0.05 | -0.73 | -0.83 | 1.1 | -0.65 | 0.56 | -0.72 | 0.87 | 0.08 | 0.36 |

*Note.* INT = intention, ATT = attitude, PBC = perceived behavioral control, IGC = intrinsic goal content, EGC = extrinsic goal content, LPA = Light physical activity,

MVPA = moderate to vigorous physical activity, M = mean, SD = standard deviation.

**p*<..05, ***p*<.01, ****p*<.001. The unit of the physical activity variables is in minute per day. Cronbach's α reliabilities are in the diagonal
